# Cases of Dysentery, Complicated with Malarious Fever

**Published:** 1871-08

**Authors:** N. S. Davis

**Affiliations:** Chicago, Ill.


### CASES OF DYSENTERY, COMPLICATED WITH MALARIOUS FEVER.—STRYCHNIA AS ONE OF THE REMEDIES, &c.

By N. S. DAVIS, M.D., Chicago, Ill.

[ Read to the Chicago Medical Society, July 24th, 1871. ]

As early as the middle of June, a severe form of dysentery began to prevail in a neighborhood in the South-west part of the West Division of the City, extending from Morgan street West to Paulina street, and from West Sixteenth street to the South Branch of the river.

The first that attracted my attention was about the 18th of June, when I was called to a family occupying the upper part of house 682 Centre Avenue, where I found two children aged about two and four years respectively, very sick with dysentery. They had been sick three or four days, and the youngest one was dying. The older one was somnolent or drowsy, with frequent paroxysms of restlessness; countenance pale and eyes sunken; skin dry; extremities cool; pulse very frequent and feeble; intestinal discharges frequent, consisting of serum and some mucus mixed with blood; stomach nauseated, with frequent efforts to vomit; respiration irregular and sighing; and general expression of great exhaustion. Some medicine was directed for him, but he died the following day. On the same day that the second child died, another boy in the same family, aged five years, was attacked with the disease, but I was not called until the day following, namely, the 21st of June. His skin was then hot and dry; his face suffused with a dark red flush; lips and mouth dry; vessels of conjunctiva red; mind somnolent, with paroxysms of restlessness; respirations hurried and irregular; pulse very rapid, small, and weak; bowels moving every few minutes, with much griping pain and tenesmus, and often accompanied by efforts to vomit. The intestinal evacuations were offensive and consisted of bloody serum and some mucus. The urine was scanty and deeper color than natural.

The rapid and extreme prostration, as indicated by the purplish redness of the face; the hurried respiration; and the frequent and feeble pulse, rendered the prognosis in this case very unfavorable. Being obliged to leave the city for a few days, the patient was left in charge of Dr. F. H. Davis, who tried faithfully the usual remedies for acute dysentery, but without any apparent control over the disease. The boy died on the 23d of June, the fourth day after the attack. These three children with their mother, had come to reside in this city only a few weeks previous to the commencement of their sickness. On the 30th of June, I was called to see W. K., an old resident of the city, laboring man, aged about fifty years, and living in the next house to the one where the three children died. He had been attacked with the dysentery about the same time that the children were sick, and during the ten days previous to my seeing him, he had received the services of two or three respectable physicians. At the time of my visit, his countenance was sunken and haggard; extremities cool and purplish; mouth and tongue very dry with active thirst; skin on trunk and face above natural temperature; abdomen concave but muscles rigid; respiration irregular, with occasional sighing; pulse 120 per minute, small and weak; very frequent intestinal discharges, consisting of serum and blood, dark grumous in appearance, and very offensive to the smell. About once a day he had a passage of nearly healthy looking fœces. He was tormented with a great sense of oppression and heat in the epigastrium, gripings and tenesmus in the bowels, and frequent efforts to vomit, which had caused the prompt rejection of almost every thing taken into his stomach. His mind was despondent and peevish. To allay the irritability of the stomach and correct in some measure the offensive quality of the intestinal discharges, we ordered the following mixture to be taken in doses of one teaspoonful every two hours:

| | | | |
|---|---|---|---|
| ℞ | Carbolic Acid Crystals | 6 grs. | |
| | Glycerine | ℥ss. | |
| | Camph. Tinct. Opii. | ℥jss. | |
| | Water | ℥ij | M. |

Also one of the following powders every four hours:

| | | | |
|---|---|---|---|
| ℞ | Tannate of Quinine | 30 grs. | |
| | Pulv. Opii | 10 grs. | M. |

Divide into six powders.

We further directed some thin wheat flour and milk porridge to be given in tablespoonful doses every half hour for nourishment. On the following day, we found our directions had been carried into effect only imperfectly, and the patient remained about the same as the previous day. We continued to attend the case until July 4th, five days in all, during which we prescribed several remedies, but no efforts on the part of myself or the family could persuade him to continue regularly any one thing long enough to gain any control over the disease, and he became daily more exhausted until he reached a fatal result. The only thing that exerted even a temporary influence, was an injection of twenty grains of pulverized ipicac, with half a grain of sulph. morph. in half a teacupful of water. This injection was used three or four times, and was each time followed by a period of two or three hours of quiet. But even these he refused to have repeated, and we were compelled to abandon the case.

The day previous to being called to the last mentioned case, we were called to a laboring man, aged 28 years, residing at the corner of Paulina and Clayton streets, about six blocks directly west of the previous cases. This man had been several months in Texas and Louisiana, and had returned only eight or ten days since. He had been taken with periodical or malarial fever on the Mississippi, while on his way here, and had been having chills and paroxysms of fever every alternate day since. He was up and dressed at the time of my visit, but presented a haggard, anemic appearance, and complained of great weakness and sense of oppression in the epigastric and hypochondriac regions. His tongue was covered with a white fur; stomach and bowels quiet; urine high colored; and sufficient increased fulness and dulness over the left hypochondriac region to indicate a moderate enlargement of the spleen. The case presenting all the characteristic symptoms of a protracted intermittent fever, he was directed the following powders:

| | | |
|---|---|---|
| ℞ Sulph. Quinine | 20 grs. | |
| Hyd. Chlid. Mite | 4 grs. | |
| Sulph. Morph | 1 gr. | M. |

Divide into four powders, to be taken at such intervals that the last would be taken at least an hour before the next expected chill. On the 30th, forty-eight hours after the first visit, an urgent message came for another visit. We found the patient in a state of extreme prostration; countenance livid; skin cool and clammy; respiration hurried and irregular; pulse frequent, small, and weak, and having frequent copious intestinal discharges consisting of a dark bloody serum, and emitting a putrid odor. I learned that he had taken all the powders ordered at the previous visit, but notwithstanding, his chill came the previous evening, which was followed by vomiting, pain in the abdomen, and frequent bloody discharges. He was directed to take a powder containing five grains of tannate of quinine, and one and a half grains of pulv. opii every two hours, and a teaspoonful of an emulsion containing twelve drops each of ol. teribenth. and tinct. opii, between. Also sinapisms over the abdomen, and astringent injections into the rectum. The remedies moderated the frequency but did not materially alter the character of the discharges, and the patient sank into complete collapse and died on the 2d July.

The day before the death of this last patient, we were called to see a woman at 692 Center Avenue, only three or four doors from the house where the three children died in one family. She was a fleshy woman about 40 years of age, who had resided there several years.

Her nursing infant had died, she said, of dysentery, and she had attended the funeral the previous day. During the night she had been attacked with severe pain in the abdomen, nausea and vomiting, with frequent discharges from the bowels, at first fæcal, but since consisting of mucus and blood.

At the time of my visit her countenance looked haggard and pinched; her skin cool and congested; pulse 118 per minute, and weak; stomach irritable, rejecting most of her drinks; intestinal discharges frequent, considerable in quantity, and mixed largely with dark blood. With the hope of allaying the irritability of the stomach, and exerting a corrective influence on the intestinal secretions, we gave this patient a teaspoonful of the following solution every two hours:

℞. Carbolic Acid Crystals.............. 6 grs.
Glycerine.............................. ℥ss.
Camph. Tinct. Opii..................... ℥ij.
Water.................................. ℥jss M.

And to check the sero-sanguinous exudation and allay the abdominal pains, a powder of tannate of quinine, 5 grains, and pulv. op. 1½ grs. was given between each of the doses of the carbolic acid solution.

On visiting the patient the next day, we found no improvement in her appearance or symptoms, except the vomiting was less frequent and the tenesmus less severe, but she was more prostrate and the intestinal discharges continuing without any change for the better. The case had every indication of progressing rapidly to a fatal termination. Being satisfied that there was a strong typho-malarial influence in all the cases I had seen in that section of the city, causing great depression in the ganglionic and vaso-motor nervous functions, and obtaining no decided effects from quinine, opium, and antiseptics, we determined to try the effects of strychnine and ipecac. We directed twenty grains of powdered ipecac to be given at once, and followed by a teaspoonful of the following solution every three hours, in sweetened water:

℞. Strychnia ---------------------------- 1 gr.

Nitric Acid ---------------------------- ʒj.

Tinct. Opii ---------------------------- ℥j.

Simple Syrup ---------------------------- ℥j.

Water ---------------------------- ℥ij. M.

At the same time my attention was called to a daughter of the patient, a girl aged about 16 years, who had been taking care of the mother, and who, during the last three or four hours, had felt frequent griping in the abdomen, some nausea, great weakness, and had three or four intestinal evacuations. Her pulse was quick and weak, skin cool and moist, and her expression dejected and anxious. She was directed to keep quiet and take one teaspoonful of the strychnine mixture directed for the mother, every four hours.

At my visit the next day, the girl was entirely comfortable, having had but one passage after she commenced taking the medicine. She took it three times a day for two days longer, and remained well.

The mother was also much better. The dose of ipecac was followed in a few minutes by free vomiting, but she retained the strychnine solution, and her intestinal discharges had been diminishing in frequency, until they do not now occur more than once in three or four hours, and contain very little blood. The pulse, the capillary circulation in the surface, and the temperature have also much improved. She was directed to continue the strychnine solution in the same doses every four hours, and limit herself to wheat flour and milk porridge for nourishment, which she did for two days more, when all symptoms of dysentery and fever had disappeared. She continued the medicine three times a day for three days longer, during which time she cautiously returned to an ordinary diet, and her recovery was complete.

On the 11th of July, we were called to Mr. A. a laboring man living at number 14 Henry street, in the same section of the city, but further north than the other cases alluded to. About two weeks since he had returned from a short sojourn in Texas and Louisiana. Soon after his return he began to feel unwell. Had lassitude, pains in his back and limbs, and indifference to food. On the 10th, he had a decided chill followed by fever and sweat, presenting the phenomena of an ordinary intermittent paroxysm. At the time of my visit at 4 o'clock P.M., of the 11th, he was presenting the initial symptoms of another chill, but nothing unusual. His tongue was covered with a whitish fur; skin cool and shrunken; pulse small but not very frequent; pains in the head, back, and limbs; but no vomiting or looseness of the bowels. Thinking the case to be one of ordinary intermittent fever, contracted, at least in part, while in the south, we directed him to have 30 grs. of sulph. quinia, 6 grs. of calomel, and 1 gr. of sulph. morphine, divided into six powders, and to take one of them every four hours. The following evening a brother of the patient called, saying that he was dying. On seeing him between 8 and 9 P.M., he really presented in a strongly marked degree the symptoms of a pernicious or congestive chill. He had taken all of the quinine powders and retained them, but about 5 o'clock P.M., the usual time for his chill, he began again to complain of chilliness and great depression, and soon became unable to speak or to swallow without great difficulty. At the time of my visit the whole cutaneous surface was cool and mottled as in extreme feebleness of capillary circulation; the face was livid; the mind extremely dull, and difficult to be roused; the pulse feeble and thready; respiration irregular, with occasional sighing; and extremities cold. We learned that he had not had much muscular trembling or shaking, but had at the commencement of the paroxysms complained of great pain in the head, back, and limbs, with a sense of depression. He had also had, during the afternoon and evening, four or five copious intestinal discharges. We directed mustard sinapisms to be applied extensively over the surface; the above mentioned solution of strychnia, nitric acid, and laudanum, to be given in teaspoonful doses every two hours until three doses had been taken, and then every three hours until otherwise directed. We also directed 20 grs. of tannate of quinia and 4 grs. of pulverized opium, to be divided into three powders, one of which was to be given at six, ten, and two o'clock the next day.

The directions were strictly followed; the stage of extreme depression gradually disappeared during the night, only one or too further intestinal discharges occurred, and the patient was comfortable during the day. At the usual time in the evening, he exhibited only slight indications of a chill, and no febrile reaction. He continued the strychnia solution every three hours for three days, when he complained of some numbness in the back of the neck, and tightness across the chest, and it was discontinued, and a pill of 2 grs. of quinia and 3 grs. of citrate of iron, ordered three times a day. His convalescence was fully established, and he has remained free from any return of chills or fever since.

We have called the attention of the Society to the foregoing cases for two purposes. First, to show that during the early and unusual warm weather during the last part of May and the first half of June, there was developed in the city, more especially in the south part of the west division, a strong typho-malarial influence, which gave rise to an unusual number of cases of severe dysentery and typho-malarial fever at an unusually early part of the season. While visiting the cases to which we have alluded, we learned that there were many other similar cases in the same section of the city, several of which proved fatal, both in children and adults. Second, to direct attention to the fact, that when we have a decided idio-miasm or typhoid influence, acting in conjunction with the ordinary malaria that produces periodical fever, we have many attacks of fever presenting distinct periodicity, but over which quinia exerts only an imperfect control. The typhoid influence seems to produce that depressed condition of the organic susceptibility and the action of the vaso-motor nervous system, which partially contra-indicates the sedative action of antiperiodic doses of quinia, but which is well remedied by efficient doses of strychnia, especially in connection with a mineral acid. We first used the combination of strychnia, nitric acid, and laudanum in the treatment of the æsthenic or type fever of dysentery, during the seasons of epidemic cholera, from 1849 to 1854, and have certainly found it very beneficial in that class of cases.

